# Multiphase aortic valve calcium scoring on true-non-contrast and calcium-preserving spectral reconstructions using dual-source photon-counting detector CT

**DOI:** 10.1007/s00330-025-11814-8

**Published:** 2025-07-22

**Authors:** Judith van der Bie, Mark M. P. van den Dorpel, Marcel van Straten, Daniel Bos, Alexander Hirsch, Nicolas M. van Mieghem, Ricardo P. J. Budde

**Affiliations:** 1 Department of Radiology & Nuclear Medicine, Erasmus MC, Rotterdam, The Netherlands; 2 Department of Cardiology, Thorax Center, Cardiovascular Institute, Erasmus University Medical Center, Rotterdam, The Netherlands; 3 Department of Epidemiology, Erasmus MC, University Medical Center Rotterdam, Rotterdam, The Netherlands

**Keywords:** Photon-counting detector CT, Computed tomography angiography, Aortic stenosis, Aortic valve calcium scores, Transcatheter aortic valve replacement

## Abstract

**Objectives:**

This study investigated differences between aortic valve calcium (AVC) scores derived from true non-contrast (TNC) and virtual-non-contrast reconstructions acquired with photon-counting detector CT (PCD-CT) and the impact of ECG-phase variability on AVC scores.

**Materials and methods:**

A hundred patients undergoing PCD-CT for transcatheter aortic valve implantation (TAVI) planning were retrospectively analyzed. Scores were computed using the Agatston methodology for TNC and virtual-non-iodine (VNI) reconstruction at scanner-selected optimal phase (best) and a fixed ECG-phase (300 ms). For VNI reconstructions, additional phases from 150 ms to 450 ms with 50 ms increments were reconstructed. AVC scores of TNC_best_ vs TNC_300_, VNI_best_ vs TNC_best,_ VNI_300_ vs TNC_300_, and all VNI phases vs VNI_best_ were compared using Wilcoxon signed-rank tests. The agreement was assessed using scatter plots, Bland–Altman plots, and intra-class coefficients. AVC scores were also categorized based on the likelihood of severe aortic stenosis. Differences between reconstructions were evaluated as percentages (reclassification) and analyzed using Cohen’s kappa coefficients.

**Results:**

TNC_best_ and TNC_300_ differed significantly (mean bias: 226; LoA: [−820, 1300]; *p* < 0.001, reclassification 17%). VNI_best_ vs TNC_best_ resulted in a mean bias of −512 (LoA: [−1900, 860]; *p* < 0.001) and reclassification of 17%. TNC_300_ vs VNI_300_ demonstrated a bias of −200 and reclassification of 14% (κ = 0.72). VNI reconstructions showed less variability across phases than the difference between TNC_best_ and TNC_300_ (range, mean bias: 22–146).

**Conclusion:**

VNI is a feasible alternative for AVC scoring but tends to overestimate compared to TNC. While phase-dependent variability in TNC underscores the need for standardization, further optimization of VNI is necessary for routine clinical use.

**Key Points:**

***Question*** *What is the performance of AVC score calculation from virtual non-contrast images with PCD-CT and the impact of the reconstructed ECG-phase*?

***Findings*** *VNI reconstructions tend to overestimate the scores compared to non-enhanced acquisitions. ECG phase significantly impacts AVC scores for non-enhanced acquisitions and VNI reconstructions*.

***Clinical relevance*** *Utilizing VNI reconstructions to calculate AVC scores might reduce radiation dose, and understanding the influence of ECG-phase on these scores might improve reliability*.

**Graphical Abstract:**

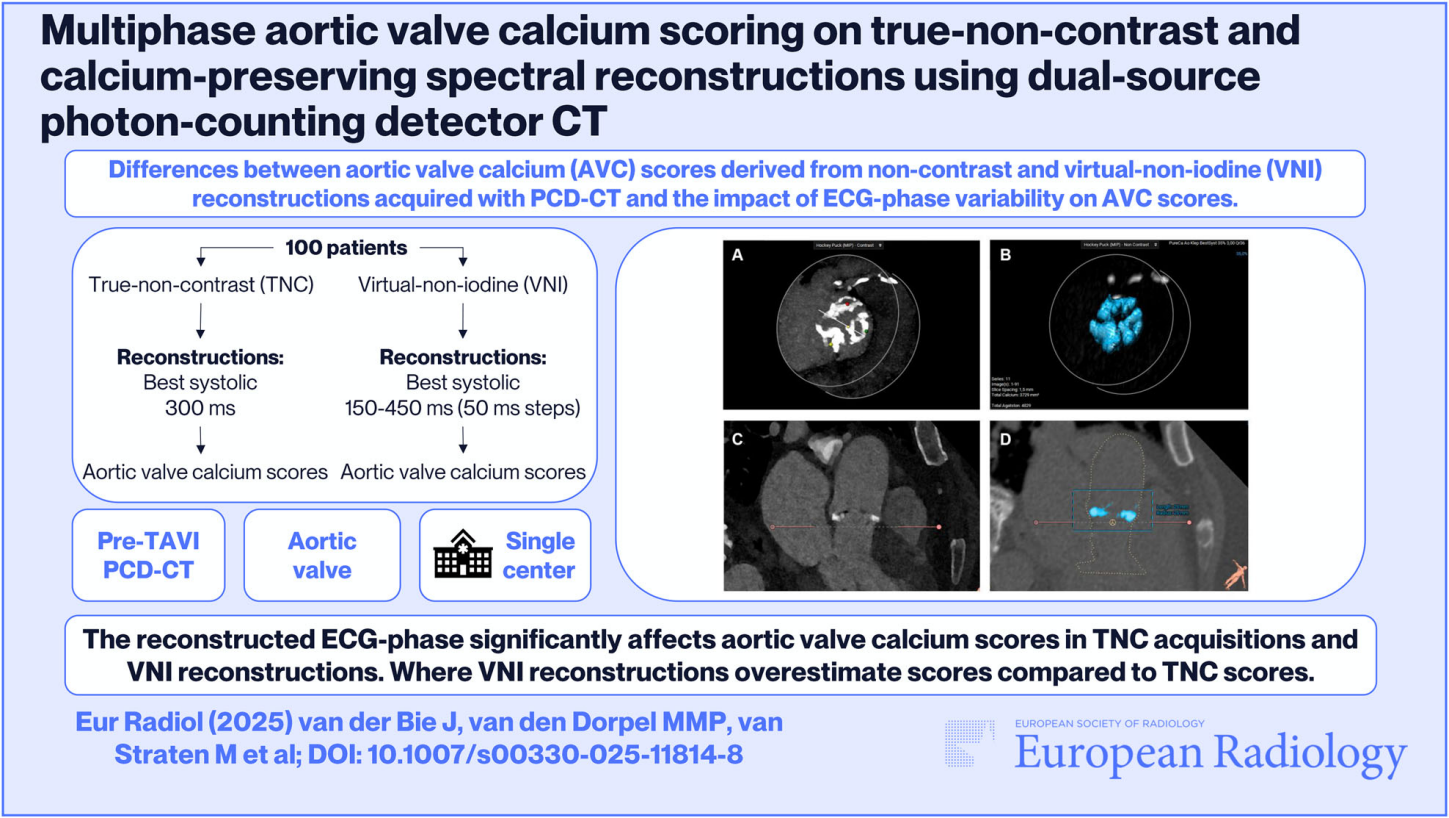

## Introduction

Aortic valve stenosis (AS) is a prevalent and clinically significant cardiovascular condition characterized by progressive narrowing of the aortic valve orifice, most often due to calcium build-up [1]. Transcatheter aortic valve implantation (TAVI) has proven superior to optimal medical therapy and non-inferior to surgical aortic valve replacement across a range of patient categories with AS [2–4]. AS is diagnosed by echocardiography, but the computed tomography-derived aortic valve calcium (AVC) score (which quantifies the extent of calcium deposition on the aortic valve) provides additional information to stratify patients with inconclusive echocardiography results while also offering prognostic value [5]. The AVC score is determined on a non-contrast CT of the heart (‘true non-contrast’, TNC) using the Agatston method [6].

CT plays an essential role in the preprocedural planning of TAVI [7]. Photon-counting detector CT (PCD-CT) encompasses the next generation of CT scanners and allows for spectral reconstructions and increased spatial resolution compared to conventional CT [8]. Spectral CT systems enable reconstruction of virtual non-contrast-enhanced images from CT angiography (CTA) acquisitions without additional scanning. PCD-CT provides two types of virtual-non-contrast (VNC) images: conventional VNC images, primarily used for iodine subtraction in soft tissues, and calcium-preserving VNC images, which allow for the visualization and quantification of calcified structures such as the aortic valve or coronary arteries [9–11]. Before the introduction of PCD-CT systems, conventional techniques such as dual-source, kV-switching, and dual-layer CT were used to generate VNC reconstructions. However, PCD-CT offers enhanced spectral separation, which may improve the accuracy of iodine subtraction and the preservation of calcified structures. Additionally, when implemented on dual-source systems, PCD-CT maintains high temporal resolution, potentially leading to more precise and diagnostically reliable VNC reconstructions. Currently, four studies investigated the performance of VNI reconstructions computed with PCD-CT for AVC score calculations [5, 12–14].

Although the Agatston method requires adherence to the end-diastolic phase (60–80% of the RR interval) for AVC score calculation, these four studies reported different reconstructed phases (Table S-1) [6, 15]. Since changes within the cardiac cycle impact both the aortic annulus geometry and the leaflet position, ECG phase variability may affect the AVC score [16, 17]. This has not previously been examined in the context of the aortic valve Agatston score, changes in the cardiac cycle have been demonstrated to affect the coronary Agatston score [18].

In this study, we examined differences between AVC scores derived from TNC and calcium preserving reconstructions and correlated the magnitude of these differences with the different ECG phases used.

## Method

### Patient selection

Consecutive adult patients (age > 18 years) who underwent a clinically indicated CT scan for TAVI planning on a dual-source PCD-CT system (NAEOTOM ALPHA, Siemens Healthineers) were retrospectively included from March to December 2023. Patients were excluded if they had undergone a previous surgical aortic valve or TAVI. Because of the observational and retrospective nature of the study, the institutional review board waived the need for ethics committee approval. General written consent was obtained from all included patients for data usage. The study adhered to the principles outlined in the Declaration of Helsinki, and it was not subject to the regulations of the Medical Research Involving Human Subjects Act, as determined by the institutional review board.

### Image acquisition and reconstruction parameters

First, a prospectively ECG-triggered (padding: 310 milliseconds (ms) from the r-wave) TNC calcium scan was acquired at 120 kV, image quality level setting of 16, and 144 × 0.4 mm collimation. Images were reconstructed at a slice thickness of 3.0 mm with 1.5 mm increments using the Qr36 kernel at 70 keV without applying iterative reconstruction. [19]. Two phases of the cardiac cycle were reconstructed: a phase deemed best by the scanner itself (in TNC_best_) and at a fixed time-point of 300 ms after the R-peak (TNC_300_).

Subsequently, a prospective ECG-triggered CT angiography (15–45% of the R–R interval) of the heart was performed, including the ascending aorta to the apex. A tube voltage of 120 kV, image quality setting of 34, and a 144 × 0.4 mm collimation were employed. The acquired scans were reconstructed with 0.4 mm slices, 0.2 mm increments at 55 keV using a Bv48 kernel and an iterative reconstruction strength of 4. The contrast protocol used was as follows: 45 mL contrast agent (Iodixanol 320), flow rate: 3.0 mL/s), was administered for the CTA of the heart. Followed by 25 mL (flow rate: 2.5 mL/s) contrast agent and a saline flush (25 mL, flow rate 2.5 mL/s) for the prospectively ECG-triggered high-pitch acquisition from the skull base to the groin.

From the CTA of the heart, calcium-preserving virtual non-contrast (VNC) images, referred to as virtual non-iodine (VNI) images, were reconstructed using the same parameters as the TNC reconstructions (70 keV, Qr36, iterative reconstruction off) [20]. VNI reconstructions utilize a manufacturer-specific algorithm (PureCalcium, Siemens) that virtually subtracts iodine-based contrast media while preserving the calcium signal. Unlike conventional VNC images, which are reconstructed from polychromatic datasets, VNI images are based on monoenergetic reconstructions and can be generated at various energy levels, improving material differentiation. In addition to identifying the best VNI reconstruction in the systolic phase (VNI_best_), images were also reconstructed at absolute delay phases ranging from 150 ms to 450 ms in 50-ms increments.

### AVC score computation

**Fig. 1:**
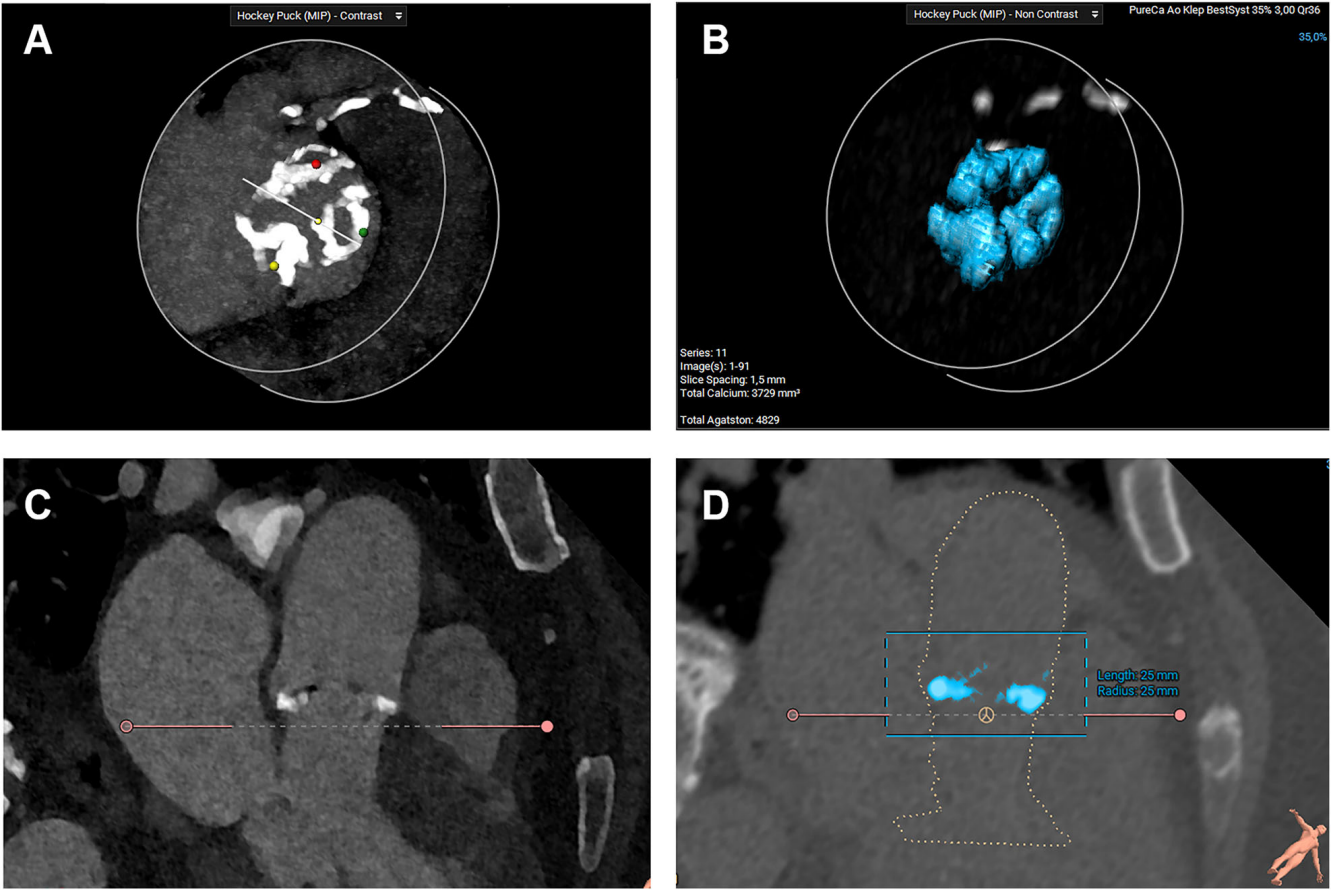
Aortic valve Agatston score computation in 3Mensio. **A** Example of aortic valve calcifications (‘hockey puck’ view). **B** Example of selected AVC in blue. All calcium with an HU ≥ 130 has been selected (‘hockey puck’ view). **C** Example of aortic valve calcifications (3-chamber view). **D** Example of selected AVC in blue. All calcium with an HU ≥ 130 has been selected (3-chamber view)

Each image series was loaded into a dedicated imaging segmentation software program (3Mensio Structural Heart, Pie Medical). First, the aortic annulus plane was obtained by marking the nadir of the 3 cusps, after which the software displayed the reference plane. Using a dedicated ‘Agatston’ module within the software, a 3D view of the aortic valve and aortic root was automatically reconstructed, displaying all areas containing calcium (≥ 130 Hounsfield Units) located between 20 mm above and 5.0 mm below the annulus. The selection of calcium areas was subsequently adjusted manually, if required (Fig. 1). All analyses were performed by a single observer with experience in calculating AVC scores (M.v.d.D., > 5 years of experience) and was blinded to the type of reconstruction/acquisition during the assessment.

### Data analysis

Categorical variables are expressed as frequencies and percentages, and continuous variables are expressed as median with 25th to 75th percentile. AVC scores were compared with a Wilcoxon signed-rank test following the following strategy. First, the AVC scores of TNC_best_ and TNC_300_. Next, AVC scores calculated from VNI_best_ and VNI_300_ were compared to TNC_best_ and TNC_300_, respectively. Lastly, AVC scores from all VNI reconstructed phases were compared to the scanner-selected best phase of VNI. Scatter plots and Bland–Altman plots supported by intra-class coefficients (ICC) were computed to assess the agreement of AVC scores as well. AVC scores of all reconstructions were divided into the following categories used to diagnose severe aortic stenosis: (a) highly likely ≥ 1600 for women ≥ 3000 for men, (b) likely ≥ 1200 for women and ≥ 2000 for men, and (c) unlikely < 800 for women and < 1600 for men [4]. Differences in category distributions were evaluated between TNC_best_ vs VNI_best_, TNC_300_ vs VNI_300_, and TNC_best_ vs TNC_300_. These differences were assessed as percentages and analyzed using Cohen’s kappa coefficients to measure agreement. All statistical analyses were performed using SPSS statistical software (IBM Corp. Released 2016. IBM SPSS Statistics for Windows, Version 28.0.1.0) and Python (Python Software Foundation. Python Language Reference, version 3.9).

## Results

### Study population

A total of 100 patients were included (57 male, 43 female), which resulted in 1000 reconstructions. Baseline characteristics and CT dose details are displayed in Table 1.

**Table 1:** Patient and scan characteristics (*n* = 100)

Age [years]	78 [76–83]
Male [%]	57%
Body mass index [kg/m^2^]	33.7 [27.3-40.0]
Heart rate [beats per min]	
True-non-contrast	72 [61–84]
CT angiography	71 [62–81]
Volume CT dose index [mGy]	
True-non-contrast	2.51 [2.1–3.1]
CT angiography	11.2 [9.0–14.7]
Dose length product [mGy cm]	
True-non-contrast	33.7 [27.3–40.0]
CT angiography	164.5 [134.3–215.8]
Size-specific dose estimate [mGy]	
True-non-contrast	3.4 [3.1–3.9]
CT angiography	14.8 [11.9–18.1]
True-non-contrast	
Phase “best” [ms]	322 [320–324]
Virtual-non-iodine	
Phase “best” [ms]	330 [304–351]

Data is presented as median [25th–75th percentile) or percentages

### AVC scores

**Fig. 2:**
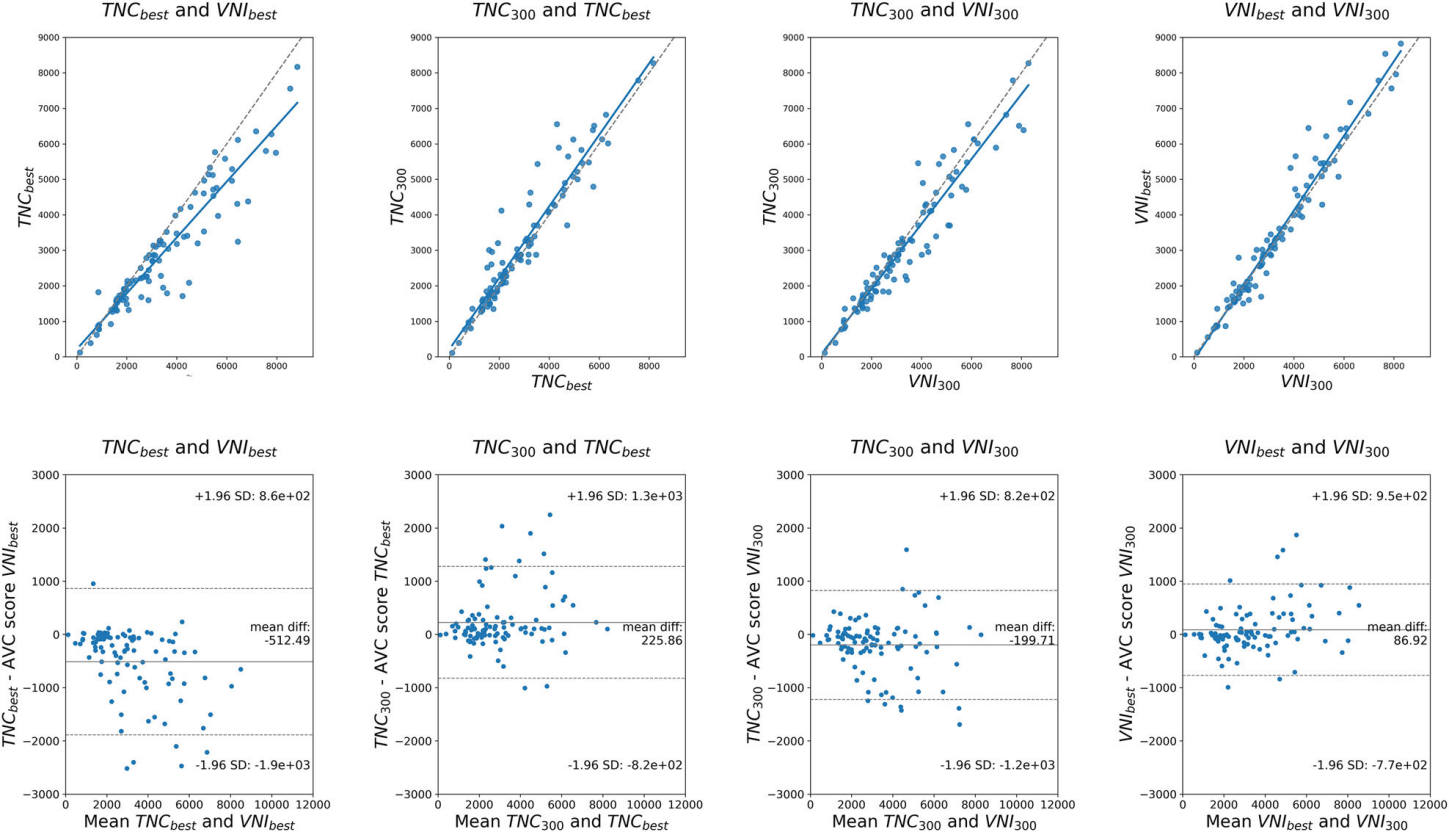
Scatter and corresponding Bland–Altman plots. VNI reconstructions overestimated AVC scores computed from TNC reconstructions. By selecting a fixed phase (300 ms) for TNC and VNI, the mean bias was reduced by approximately 50%. Examining the variation between phases, the bias for TNC_best_ and TNC_300_ is roughly 65% higher than for VNI_best_ compared to VNI_300_

A median AVC score of 2364 [1633–3977] was observed for TNC_best_ compared to 2743 [1765–4286] for TNC_300_ (Table 2). Despite a time difference of only 22 ms, these scores were significantly different (Fig. 2: mean bias: 226, LoA: [−820:1300], *p* < 0.001). The direct comparison between TNC_best_ (2364 [1633–3977]) vs VNI_best_ (3018 [1815–4804]) also showed significantly different AVC scores (*p* < 0.001, Table 3). VNI_best_ overestimated AVC scores compared to TNC_best_, with a mean bias of −512 (LoA: −1900; 860) (Fig. 2). AVC scores differed significantly between TNC300 (2743 [1765–4286]) and VNI300 (2946 [1884–4685]), with a mean bias of −200 and LoA of [−1200; 820].

**Table 2:** Median AVC scores per reconstruction compared to TNC_best_

	Median calcium score	Mean difference (%)
TNC_best_	2364 [1633–3977]	REF
TNC_300_	2743 [1765–4286]	+10% (±21%)
VNI_best_	3018 [1815–4804]	+19% (±28%)
VNI_150_	3006 [1858–4661]	+17% (±29%)
VNI_200_	2900 [1846–4561]	+20% (±29%)
VNI_250_	2984 [1828–4478]	+21% (±29%)
VNI_300_	2946 [1884–4685]	+18% (±27%)
VNI_350_	2948 [1789–4444]	+17% (±28%)
VNI_400_	2895 [1705–4422]	+15% (±28%)
VNI_450_	2901 [1746–4400]	+16% (±28%)

*AVC* aortic valve calcium, *TNC* true-non-contrast, *VNI* virtual-non-iodine

**Table 3:** *p*-values of Wilcoxon signed rank tests (white) and ICCs (gray) per reconstruction

*ICC* intra-class correlation coefficient, *TNC* true-non-contrast, *VNI* virtual-non-iodine

### Variability of AVC scores

**Fig. 3:**
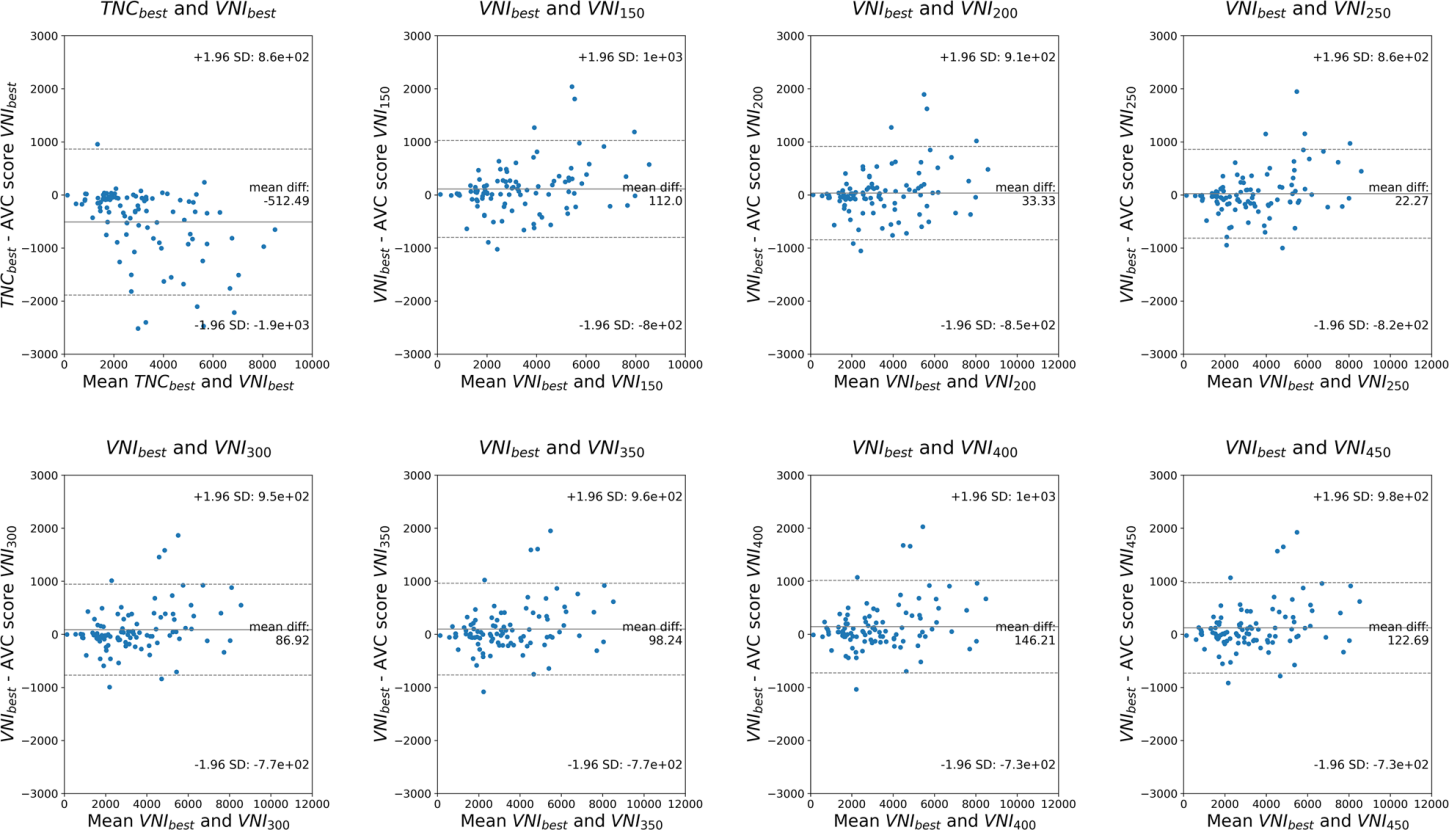
Blant-Altman plots illustrate comparisons between VNI_best_ and all other VNI reconstructions

The variability of AVC scores was assessed by comparing scores computed from VNI_best_ (median *t* = 326 ms) with the obtained different phases of the VNI reconstructions as illustrated in Fig. 3. Mean biases ranged from 22 (VNI_250ms_) to 146 (VNI_400ms_). VNI_150,_ VNI_250ms_, VNI_300ms_, and VNI_350ms_ demonstrated similar calcium scores to VNI_best_ (*p* > 0.05).

### Reclassification of AS severity

**Fig. 4:**
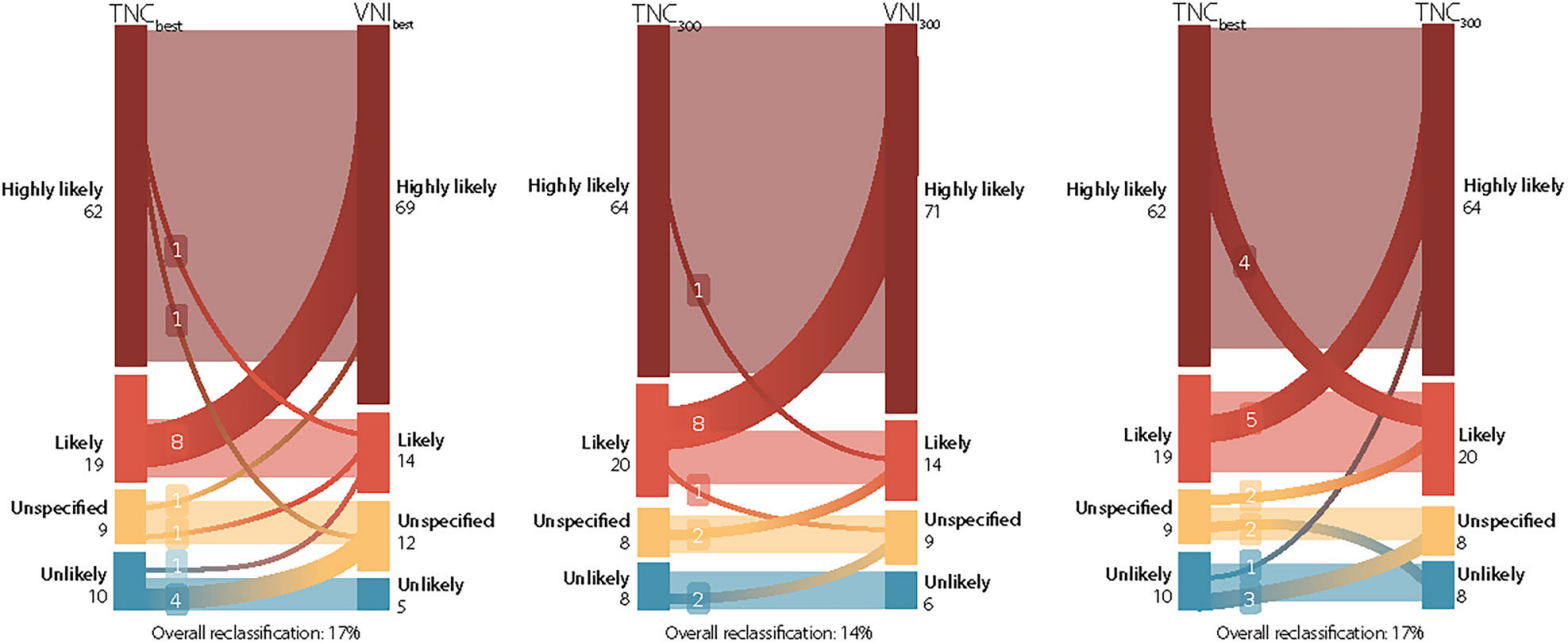
Blue is unlikely severe AS (< 800 for women and < 1600 for men), yellow is unspecified (800–1200 for woman and 1600–2000 for men), light red is likely severe AS (≥ 1200 for women and ≥ 2000 for men) and dark red is highly likely severe AS (≥ 1600 for women ≥ 3000 for men). Both TNC_best_ to VNI_best_/TNC_300_ showed a misclassification rate of 17% of the patients. For VNI reconstruction, patients were more often classified in a higher risk group. For all comparisons, the largest shift was seen in the “likely severe AS” group

Between TNC_best_ to TNC_300_, 17 (17%, κ = 0.69) patients with a median difference of −201 [−378; 142] were assigned to a new class (Fig. 4). Again, 17 (17%, κ = 0.66) patients were reclassified when comparing AVC scores from TNC_best_ to VNI_best_. Among the misclassified patients, the median difference in AVC scores for misclassified patients was −303 [−753; −75]. The lowest reclassification rate was obtained with TNC_300_ to VNI_300_, with 14 (14%, κ = 0.72) of the patients reclassified with a median difference of 281 [−790; −206]. For the various phases of VNI compared to VNI_best_, the reclassification rate ranged from 8% at 350 ms to 18% at 150 ms. The lowest overall reclassification rate, at 3%, occurred between VNI_350_ and VNI_450_.

## Discussion

Our study compared AVC scores of TNC images to VNI images reconstructed from PCD-CT scans and AVC scores computed from multiple reconstructed phases of the heart cycle. The most accurate AVC scores were observed using a fixed phase of 300 ms for both TNC and VNI reconstructions. Compared to TNC, VNI consistently overestimated AVC scores, with a mean bias of −200 and a reclassification rate of 14% (κ = 0.72). When using scanner-selected optimal phases, VNI reconstructions overestimated (mean bias: −512) AVC scores obtained from TNC reconstructions. TNC reconstructions with a minimal time difference, also demonstrated a significant difference in AVC scores with an equal number of patients reclassified as TNC/VNI optimal phases. Indicating AVC scores substantially vary within scans from the same acquisition if the reconstructed phase is different. VNI reconstructions showed less variability of AVC scores over the reconstructed phases.

Four studies investigated the feasibility of AVC scoring with PCD-CT compared to TNC acquisitions (Table S-1). All studies employed standard Agatston reconstruction, similar to the approach in the present study. Feldle et al investigate VNI reconstructions acquired with prospective high-pitch and retrospective acquisitions [14]. AVC scores of VNI and TNC demonstrated high correlations, however, no exact scores of VNI were reported. Diagnostic accuracy was investigated only by categorizing patients in severe or non-severe AS, leading to 85% diagnostic accuracy in high pitch mode and 81% with retrospective gating.

Risch et al investigated AVC scores with standard Agatston reconstruction and reconstructions with thinner slice thicknesses and found an underestimation of AVC scores computed with VNI to TNC from −25% up to −32% [12]. The smallest difference was achieved with thin slices (0.4 mm) and an iterative reconstruction strength of four. We found a smaller difference of +19% but an overestimation. This difference might be explained by the use of high-pitch acquisitions and the difference in the reconstructed phase.

In addition to the standard Agatston reconstruction at 70 keV, Mergen et al generated multiple virtual monoenergetic images at 60 keV, 70 keV, 80 keV, and 90 keV, and utilized iterative reconstruction with strengths of two, three, and four to reconstruct VNI images computed from cardiac late enhancement scans. Mean difference biases ranged from 258 to 267 at 70 keV, which were lower than our study; however, they also indicated a significant overestimation of AVC scores. Mergen et al reconstructed the scans at 280 ms relative to the R-wave, which could account for the discrepancies observed in comparison to our study.

Additionally, Mergen et al explained that residual iodine might be in the center of macro calcifications even after using the VNI algorithm to subtract iodine from the image. This phenomenon may also have contributed to the overestimation of AVC by VNI compared to TNC in our study. Some voxels containing iodine were mistakenly identified as true calcium voxels due to the simple counting method used in the Agatston score. By increasing the keV levels to 80 keV, results similar to those obtained by TNC acquisition were achieved, with mean bias differences ranging from 3 to 11. Switching to VNI images at 80 keV might alter the contribution of pixels to the score, but rather slightly reduce the total score. This reduction is due to how the Agatston score is calculated, which places more weight on higher attenuation values. Utilizing higher KeV reconstructions does not improve the performance of the VNI algorithm, it only adjusts the score by a different weighting. Because Agatston scoring is validated at 120 kV (corresponding to ~70 keV), and algorithms are optimized for this energy range, we recommend reconstructing VNI images at 70 keV [21, 22]. However, even in the absence of visible iodine, slight overestimation was observed compared to non-enhanced scans. This indicates that inter-scan and intra-scan variability, arising from factors such as patient positioning and cardiac motion, may also play a role [23].

Sartoretti et al investigated the influence of temporal resolution on AVC scores and found that lower temporal resolution leads to the overestimation of AVC scores caused by motion artifacts and blurring of calcifications [13]. In our study, we utilized a high temporal resolution of 66 ms, which helped minimize motion artefacts and blurring that may lead to overestimation of the AVC score. Nonetheless, we observed noticeable variability in TNC-derived AVC scores across different cardiac phases, even when reconstructed at the same energy level and with identical parameters. This suggests that AVC quantification is inherently sensitive to the timing of image acquisition within the cardiac cycle, and that variability can persist despite high temporal resolution [23].

There are several limitations to this study. First, it is retrospective and conducted at a single center, involving a restricted number of patients. Thus, the findings may not fully represent the broader population. Secondly, to limit our data, we selected one additional phase for 300 ms for the non-enhanced scans. As a result, we could only perform a single comparison of variability. This may introduce bias to our study, as we reconstructed multiple phases for VNI. However, we observed a significant difference between the two phases of TNC, emphasizing the importance of standardization, which is the central message of our manuscript. Third, this study only investigated the Agatston reconstruction method with a 3.0 mm slice thickness. Risch et al showed improved results with thin slices (0.4 mm) and a high iterative reconstruction strength of four due to the sharp delineation of calcifications. Employing reconstructions with higher spatial resolution might aid the performance of VNI reconstructions in computing AVC scores. Fourth, like Mergen et al, relatively high heart rates (median: 71 beats per min TNC, median: 72 beats per min VNI) were included in this study. Lower heart rates may lead to more accurate calcium quantification on both TNC and VNI reconstructions. However, Beta-blockers cannot be administered preventively in this patient population. Finally, no outcome measures were available in the current study to examine the prognostic value of both VNI- and TNC-derived AVC scores. Follow-up studies may aim to examine potential differences in the prognostic value of the different types of reconstructions.

In conclusion, VNI reconstructions are feasible for computing AVC scores, but they demonstrated an overestimation compared to AVC scores computed from TNC reconstructions. VNI reconstructions showed less variation between reconstructed phases than TNC reconstructions. However, AVC-scoring is not very robust since only a small change in ECG-phase results in a significant change in score. Fixed phases demonstrated the smallest difference (mean bias = −200) between the two reconstructions and the lowest reclassification of 14%, which may be related to less anatomic variation due to less motion difference between the scans. Therefore, standardization is needed to determine at which phase TNC or VNI reconstruction should be performed to enable comparison of AVC scores. Further improvements are required to implement the VNI reconstruction algorithm in daily clinical practice.

## ELECTRONIC SUPPLEMENTARY MATERIAL

ELECTRONIC SUPPLEMENTARY MATERIAL

## Abbreviations

AS: Aortic valve stenosis
AVC: Aorta valve calcium
CTA: Computed tomography angiography
PCD-CT: Photon-counting detector CT
TAVI: Transcatheter aortic valve implantation
TNC: True non-contrast
VNC: Virtual-non-contrast
VNI: Virtual-non-iodine
