# Supplementary material for: Multiphase aortic valve calcium scoring on true-non-contrast and calcium-preserving spectral reconstructions using dual-source photon-counting detector CT
Source: Eur Radiol. 2025 Jul 22;36(1):194–202. doi: 10.1007/s00330-025-11814-8 (PMC12711920; doi:10.1007/s00330-025-11814-8)
Supplement: Supplementary file 1 — ELECTRONIC SUPPLEMENTARY MATERIAL [file 330_2025_11814_MOESM1_ESM.pdf]

Multiphase aortic valve calcium scoring on true-non-contrast and calcium-preserving spectral reconstructions using dual-source photon-counting detector CT

ELECTRONIC SUPPLEMENTARY MATERIAL

Table S-1 Scan protocols

	**Van der Bie et al.***	**Feldle et al [14].**	**Mergen et al [5].**	**Risch et al [12].**	**Sartoretti et al [13].**
**CT system**	NAEOTOM ALPHA	NAEOTOM ALPHA	NAEOTOM ALPHA	NAEOTOM ALPHA	NAEOTOM ALPHA
**Patients (n)**	100	150	90	41	66
**Tube voltage [kV]**					
TNC	120	120	120	120	120
VNI	120	120	120	120	120
**Collimation [mm]**	144x0.4	144x0.4	144x0.4	144x0.4	NR*
**Reconstructed slice thickness [mm]**					
TNC	3.0	3.0	3.0	3.0	3.0
VNI	3.0	3.0	3.0	1.0<br>0.4	3.0
**Increments [mm]**					
TNC	1.5	1.5	1.5	1.5	1.5
VNI	1.5	1.5	1.5	0.4<br>0.2	1.5
**Scan mode**					
TNC	Prospective sequential	Prospective high pitch	Prospective sequential	Prospective high pitch	Prospective sequential
VNI	Prospective sequential	Prospective high pitch OR Retrospective variable**	Prospective sequential	Prospective high pitch	Prospective sequential
**Phase**	Best systolic; 300ms, +150;:50:450ms	NR	230 ms from R-wave	NR	280 ms from R-wave
**Matrix size**	512x512	512x512	512x512	512x512	512x512
**Virtual monoenergetic image [KeV]**					
TNC	70	70	70	70	70
VNI	70	70	60, 70, 80, 90	70	70
**Kernel**					
TNC	Qr36	Qr36	Qr36	Qr36	Qr36
VNI	Qr36	Qr36	Qr36	Qr36, Br36	Qr36
**Iterative reconstruction strength**					
TNC	Off	Off	Off	Off	Off
VNI	Off	Off	2,3,4	4	Off

*Current study
**Depending on the heart rate

NR; not reported, TNC; True non-contrast, VNI; virtual-non-iodine
